# Enzymatic and Nonenzymatic Antioxidant Systems Affected by the Application of Hydrothermal Treatment and Calcium Chloride in Papaya Fruit

**DOI:** 10.1155/ijfo/9794917

**Published:** 2026-04-30

**Authors:** Blanca Alicia López-Zazueta, Martha Edith López-López, Roberto Gutiérrez-Dorado, Jordi Gerardo López-Velázquez, Denisse Aurora Díaz-Corona, Evangelina García-Armenta, Misael Odin Vega-García, Lidia Elena Ayón-Reyna

**Affiliations:** ^1^ Graduate Program in Food Science and Technology, Faculty of Chemical and Biological Sciences, Autonomous University of Sinaloa, Americas Avenue and Josefa Ortiz Without Number University City, Culiacan, 80010, Sinaloa, Mexico, unam.mx; ^2^ Technological University of Culiacan, Culiacan-Imala Highway Km. 2, Culiacan, 80014, Sinaloa, Mexico

**Keywords:** calcium salt, *Carica papaya*, hot water treatment, oxidative stress, postharvest quality

## Abstract

Papaya fruits have a high content of bioactive compounds that provide them high antioxidant capacity; however, these fruits undergo rapid deterioration processes once harvested, which implies a decrease in antioxidant activity. In order to reduce the oxidative stress that causes deterioration in the fruit, it is important to use technologies to increase the antioxidant system of the fruit. In that sense, the aim of this work was to evaluate the effect of a hydrothermal treatment (HT), calcium chloride (CaCl_2_), and their combination (HT‐CaCl_2_) on the physicochemical quality and the enzymatic and nonenzymatic antioxidant systems of papaya fruits. Harvested papaya was treated with HT (48°C, 25 min), CaCl_2_ (1% w/v), and the combination HT‐CaCl_2_ followed by storage at 12°C for 20 days. Physicochemical quality (weight loss, external color, and firmness), bioactive compounds (ascorbic acid [AA], total phenolics, and carotenoid content), antioxidant capacity (ABTS and DPPH), and the activity of antioxidant system enzymes (peroxidase [POD] and ascorbate peroxidase [APX]) were evaluated. In this regard, HT‐CaCl_2_ was the most effective treatment in controlling weight loss, preserving the external fruit color, and maintaining the firmness. Furthermore, HT‐CaCl_2_ boosted the fruit antioxidant properties by maintaining the amount of ascorbic acid and increasing total phenolics and carotenoids. Thus, fruits treated with HT‐CaCl_2_ showed a higher antioxidant capacity as well as increased activity of the enzymes of the antioxidant system. In general, HT‐CaCl_2_ can be used as an effective treatment to enhance antioxidant properties by maintaining bioactive compounds and increasing antioxidant enzymes, as well as maintaining the physicochemical quality of papaya fruits.

## 1. Introduction

Papaya (*Carica papaya* L.) is a native fruit from Central America and, due to its organoleptic characteristics and high content of bioactive compounds, such as ascorbic acid (AA), phenolics, and carotenoids, is considered one of the most important crops worldwide. However, papaya fruit is subjected to extensive postharvest losses through accelerated ripening and senescence [1, 2]. During these stages, a deterioration of the cell membrane occurs, producing a vast number of highly reactive molecules called reactive oxygen species (ROS), which cause oxidative stress and tissue catabolism [2–4].

Oxidative stress occurs when the formation rate of ROS is greater than the removal rate, which makes the system unable to return to homeostasis. Also, ROS begin to degrade proteins, induce DNA mutations, and peroxidize lipids, which significantly reduces the nutritional and physicochemical quality, resulting in a short shelf life [4]. Therefore, it is necessary to implement technologies to reduce stress and the accelerated senescence.

Treatments, such as hydrothermal treatment (HT) and calcium chloride (CaCl_2_), have been implemented on horticultural products to preserve postharvest quality, bioactive compounds, and increase antioxidant properties [5]. It has been reported that the oxidative stress during fruit postharvest is decreased by HT application due to activation of the enzymatic and nonenzymatic antioxidant mechanisms to eliminate ROS, such as superoxide anion and hydrogen peroxide. Respecting nonenzymatic antioxidant activity, HT increases the content of antioxidant compounds such as phenolics, carotenoids, and AA, which eliminate oxy radicals and singlet oxygen [6]. Likewise, HT induces the enzymatic antioxidant activity by activation of antioxidant enzymes such as peroxidase (POD) (catabolizes H_2_O_2_ in water) and ascorbate peroxidase (APX) (catalyzes H_2_O_2_‐dependent oxidation) [7]. On the other hand, CaCl_2_ is an essential nutrient that binds to the cell membrane surface and prevents ROS‐induced oxidation of lipids, maintaining the integrity of the membrane. Also, Ca^+2^ avoids weight loss by opening and closing stomata, providing firmness and turgidity to the fruit [8]. The beneficial effects of HT and CaCl_2_ on fruit quality have been demonstrated individually; nevertheless, a synergistic effect has been observed when the treatments are applied in combination because HT favors a greater formation of calcium pectates due to the fact that heat activates enzymes and generates available places on galacturonic acids for calcium to bind; therefore, it prevents tissue deterioration, delaying the normal ripening and senescence [5]. Also, heat facilitates calcium diffusion into fruit tissues, while calcium stabilizes cell structures and delays senescence‐related changes. HTs can reduce microbial load, delay ripening processes, and induce stress responses that stimulate the synthesis or retention of protective metabolites. Meanwhile, calcium plays a key structural role in maintaining cell wall integrity by forming cross‐links with pectic polysaccharides in the middle lamella, thereby improving tissue firmness and reducing membrane permeability [5, 6]. Consequently, this combined strategy has gained attention as a promising approach to improve fruit quality, maintain antioxidant compounds, and extend postharvest shelf life.

Some studies have reported the combined effect of HT with different calcium salts on postharvest quality, chilling injury, control of pathogenic diseases, nutritional content, antioxidant capacity, cell wall enzymes, among others, in whole fruits such as mango [9], kiwifruit [10], and strawberry [11]; and in fresh‐cut fruits such as papaya [12], melon [13], and apple [6, 14]. However, there are no studies that have evaluated the combination of an HT with CaCl_2_ in harvested papaya fruits in order to improve resistance to oxidative stress (enzymatic and nonenzymatic antioxidant system) and in this way maintain the organoleptic quality of the fruit and increase its shelf life. Therefore, there is interest in observing the effect of these treatments on the bioactive compounds, antioxidant capacity, and activity of antioxidant system enzymes of papaya fruit, as well as verifying their effect on the physicochemical quality of the fruits.

## 2. Materials and Methods

### 2.1. Materials

Papaya fruit cv. Maradol was harvested at maturity index 2, which corresponds with fruit of green skin with a well‐defined yellow stripe; pulp that is orange in color near the seed cavity and light green near the skin, although still hard (65–68 N); and an average value of total soluble solids of 7.5°Brix [15] in a commercial plantation near Culiacan, Sinaloa, Mexico (located at 24°52′056.800″ N latitude and 107°26′052.100″ W longitude). Fruits were selected based on the absence of defects, bumps, bruises, and damage by microorganisms, ensuring color and weight uniformity. Food‐grade calcium chloride (CCFO21‐00 Fabpsa, Mexico) was used.

### 2.2. Treatment Applications

Papaya fruits were washed with tap water followed by sanitizing with a sodium hypochlorite solution (1% v/v) for 5 min and left at room temperature for 1 h to remove excessive moisture. Fruits were randomly divided into 4 groups for treatment application. The first group was immersed in purified water at 25°C (control); the second group was immersed in a calcium chloride solution (1% w/v) at 25°C (CaCl_2_) [16]; the third group was hydrothermally treated by immersion of the fruits in purified water at 48°C (HT) using a water bath model 1266‐02 (Cole Parmer, Vernon Hills, Illinois, USA) according to Ayón‐Reyna et al. [16]; and the last group was treated with the combination HT‐CaCl_2_ using the conditions already described. All treatments had an immersion time of 20 min. Fruits were stored at 12°C with a relative humidity of 90%–95% for 20 days. Three fruits/treatments/replicates were randomly removed every 4 days to perform the analyses. Three replicates were carried out, and each replicate consisted of 72 fruits.

### 2.3. Postharvest Quality Analysis

#### 2.3.1. Weight Loss

The fruit weight was recorded every 4 days using a Sartorius model TE 4101 balance (Goettingen, Germany). The values were expressed as a percentage of weight loss in relation to the initial weight [17].

#### 2.3.2. External Color

Fruit color was measured in the equatorial region using a CR‐200 colorimeter (Minolta Co. Ltd., Osaka, Japan), and CIELAB color parameters (*L* ^∗^, H°) were recorded as the means of nine measurements per treatment [1].

#### 2.3.3. Firmness

Pulp firmness was determined using a digital penetrometer (Chatillon DFE 100; AMETEK Inc., Largo, FL, USA) equipped with an 11 mm diameter flat tip, at a penetration depth of 5 mm with a speed of 50 mm/min. The results were expressed in Newtons [14].

### 2.4. Bioactive Compounds

#### 2.4.1. AA

This study uses the method of López‐Zazueta et al. [2], and the methods description partly reproduces their wording. Approximately 10 g of frozen pulp were obtained from each fruit and homogenized in 50 mL of cold, deionized, and degassed water using a blender (Osterizer, Jarden Corp., Rye, NY, USA). The homogenate was filtered through several layers of organza to remove solids and then passed through 0.45 μm membrane filters (Pall Corp., Port Washington, NY, USA). To remove pigments and interfering substances, the clarified extract was further purified using Sep‐Pak C18 cartridges (Waters Corp., Milford, MA, USA). A 1 mL aliquot of the purified extract was injected into an HPLC system (Agilent 1100 Series, Waldbronn, Germany) equipped with a reversed‐phase column (Sphereclone ODS2, 250 mm × 4.6 mm, 5 μm particle size; Phenomenex, Torrance, CA, USA) maintained at 16°C. The chromatographic separation was achieved using a 25 mmol/L monobasic potassium phosphate buffer as the mobile phase, operating at a flow rate of 0.7 mL/min. The elution was monitored at 254 nm with a 10 μL injection volume and a total run time of 15 min. Quantification of AA was performed by external calibration using standard solutions of L‐AA (Sigma‐Aldrich, St. Louis, MO, USA). The concentration of AA was expressed as milligrams per 100 g of fresh weight (mg AA/100 g FW). Nine repetitions were made for each treatment.

#### 2.4.2. Total Phenolics (TP)

This study was conducted using the Folin–Ciocalteu colorimetric method, with adjustments based on the procedures previously described by Moo‐Huchin et al. [18] and López‐Zazueta et al. [2]. Approximately 5 g of frozen pulp were mixed with 10 mL of methanol and homogenized with an Ultra‐Turrax homogenizer (Model T18 basic, IKA, Germany). The resulting slurry was sonicated (30 min) and centrifuged at 3000 × *g* (10 min, 4°C) (Eppendorf 5804‐R, Germany). The obtained supernatant was collected, and the residue was extracted again under identical conditions to maximize recovery of phenolic compounds. Both supernatants were combined, the solvent was evaporated, and the extract was reconstituted to 4 mL with methanol. For the assay, 40 μL of the methanolic extract (1:10 v/v dilution) was mixed with 360 μL of Folin–Ciocalteu reagent (diluted 1:8 v/v) and 100 μL of 7% Na_2_CO_3_ solution. After incubation for 90 min at 21°C in the dark, absorbance was measured at 765 nm using a microplate spectrophotometer (Synergy HT, BioTek Instruments, Winooski, VT, USA). A calibration curve was constructed with gallic acid (GA) standards (50–600 μg/mL), and results were reported in mg GA equivalents per 100 g of fresh weight (mg GAE/100 g FW).

#### 2.4.3. Total Carotenoids (TC)

The determination was made using the technique described by López‐Valenzuela et al. [19] with some modifications. For the extraction, 10 g of sample was homogenized with 10 mL of acetone for 1 min in an Ultra‐Turrax (IKA T18 basic, IKA, Germany) and then filtered using organza cloth. The filtrate was discarded, and the pellet was recovered and homogenized with 10 mL of acetone for 1 min and filtered again. In this case, both the filtrate and the pellet were recovered; the pellet was homogenized again with 10 mL of acetone and filtered on organza cloth. The obtained filtrate was mixed with the previously recovered filtrate and passed through 0.45 μm syringe filters (HPLC‐certified PVDF membrane, Pall, USA). A 1 mL aliquot was taken and analyzed on HPLC equipment. A 250 mm × 4.6 mm × 5 μm carotenoid YMC column was used, with a temperature of 30°C; a mobile phase of tert‐butyl methyl ether (15%), methanol (81%), and water (4%) until achieving a gradient at 75 min of 77.5% tert‐butyl methyl ether, 18.5% methanol, and 4% water; a flow of 0.7 mL/min until 45 min to reach a flow of 0.8 mL/min at 75 min; a wavelength of 447 nm; an injection of 100 μL; and a run time of 75 min. The carotenoid content was obtained using a calibration curve of a pure standard, and the results were expressed as mg β‐carotene/100 g FW.

### 2.5. Determination of Antioxidant Activity

#### 2.5.1. ABTS (2,2′‐Azino‐Bis(3‐Ethylbenzothiazolin‐6‐Sulfonic)) Method

The antioxidant capacity was assessed using the ABTS^+^ radical discoloration method, following the protocol adapted for papaya extracts described by López‐Zazueta et al. [2]. The ABTS^•+^ radical cation was generated by reacting 7 mM ABTS solution (5 mL) with 140 mM potassium persulfate (88 μL) and allowing the mixture to stand for 12–16 h at room temperature in darkness. Before analysis, the stock solution was diluted with phosphate‐buffered saline (PBS, 7 mM, pH 7.4) until an absorbance of 0.75 ± 0.02 was reached at 734 nm. For the assay, 3 μL of the methanolic extract (obtained previously for the analysis of TP and diluted 1:18, w/v) were mixed with 197 μL of ABTS solution in microplate wells. The mixture was incubated at 27°C for 30 min under dark conditions, and absorbance was then recorded at 734 nm using a microplate reader (Synergy HT, BioTek Instruments, Winooski, VT, USA). Trolox was used as the reference antioxidant to prepare the calibration curve (0–225 μg/mL). Results were reported as micromoles of Trolox equivalents (TE) per 100 g of fresh weight (μmol TE/100 g FW).

#### 2.5.2. DPPH (2,2‐Diphenyl‐1‐Picrylhydrazyl) Method

The free radical scavenging activity of papaya extracts was evaluated using the DPPH method with the procedure reported by López‐Zazueta et al. [2]. Briefly, 20 μL of the diluted methanolic extract (1:2 v/v) were combined with 180 μL of a 150 mM methanolic DPPH solution in microplate wells. The mixture was incubated for 30 min at 27°C in complete darkness to allow the reaction to reach equilibrium. After incubation, absorbance was measured at 550 nm using a microplate spectrophotometer (SynergyTM HT Multi‐Detection, Biotek, Inc., Winooski, VT, USA). Trolox (0–225 μg/mL) was employed as the standard for calibration (Sigma‐Aldrich‐238813, St. Louis, MO, USA), and the antioxidant activity of the samples was calculated from the Trolox standard curve. The results were expressed as μmol TE/100 g FW.

### 2.6. Enzyme Extraction

The enzyme extraction was performed as previously described by Díaz‐Corona et al. [5] with some modifications. Frozen papaya pericarp (5 g), obtained from each fruit, was homogenized with 5 mL of extraction buffer containing 25 mM sodium borate (pH 8.8), 1 mM EDTA, and 5% polyvinylpyrrolidone. The homogenate was centrifuged at 18000 × *g* for 40 min at 4°C, and the obtained supernatant was filtered on organza cloth and stored at −70°C until the enzymatic test was performed. For the determination of enzymatic activity, the protein content was evaluated according to the Bradford [20] method, using bovine serum albumin as a calibration standard at 595 nm.

### 2.7. Enzyme Activity

#### 2.7.1. POD

POD activity was determined according to López‐López et al. [14] with some modifications. For the quantification of the activity, 2750 μL of phosphate buffer (1 M, pH 7.8), 100 μL of guaiacol (1% v/v), 100 μL of H_2_O_2_ (0.46% v/v), and 50 μL of the enzyme extract were mixed and read in a spectrophotometer (UNICO SQ 2800, NJ, USA). The specific activity of POD was expressed as unit of activity (UA)/mg of protein, where U was defined as the change in absorbance of 0.001 units/min.

#### 2.7.2. APX

APX activity was determined following the methodology of Díaz‐Corona et al. [5] with some modifications. The reaction mixture contained 969 μL of potassium phosphate buffer (40 mM, pH 7.0), 5 μL of L‐AA (0.1 M), 1 μL of H_2_O_2_ (0.1 M), and 25 μL of the enzyme extract. AA concentration was calculated based on the extinction coefficient (2.8/mM·cm) at 290 nm, and the activity was expressed as UA/mg of protein.

### 2.8. Statistical Analysis

The experiment was conducted using a completely randomized design, considering treatment and storage time as factors. Fruits were randomly assigned to each treatment, with three independent biological replicates and three repetitions. Each replicate consisted of 72 fruits, resulting in a total of 18 fruits per treatment per replicate. Statistical analysis of data was performed through analysis of variance using the software Statgraphics Plus 5.1 (Statistical Graphics, Warrenton, VA, USA), and the means were compared using Fisher’s least significant difference (LSD) test at a significance level of *p* ≤ 0.05. The means with their corresponding standard deviation and letters indicating LSD at each time point were plotted using SigmaPlot 12.0 (SYSTAT Software Inc., San Jose, CA, USA).

## 3. Results and Discussion

### 3.1. Postharvest Quality Analysis

#### 3.1.1. Weight Loss

Weight loss percentage increased during storage, obtaining values between 10.48 and 13.67 after 20 days at 12°C and presenting significant differences only on the last day of evaluation between control and HT‐CaCl_2_ treatments (Table 1). The results showed that HT‐CaCl_2_‐treated fruits exhibited the lowest weight loss, showing a synergistic effect of the treatments since individually HT and CaCl_2_ were not as effective. According to Correa et al. [21], the application of calcium salts under high‐temperature conditions enhances calcium diffusion through the porous apoplast and promotes its retention in the cell wall. Additionally, heat induces changes in gene expression and protein synthesis during fruit ripening. These modifications inhibit ethylene production and reduce the activity of enzymes responsible for cell wall degradation. High‐temperature treatments also increase the respiration rate, likely as a response to heat stress experienced by the fruit. Likewise, HT exerts a direct effect on the epicuticular waxes of papaya fruits, which, as they become more fluid, cover the microwounds and stomata, reducing gas exchange and water vapor release [16]. In addition to this, calcium ions are involved in stomatal opening and closing; if the calcium concentration around the occlusive cells increases, the stomatal conductance decreases and promotes the closure of the stomata, avoiding water loss [22]. These results are consistent with those reported by Naser et al. [17], Shafiee et al. [11], Castellano et al. [23], and Torres et al. [24], who pointed out that the combined application of an HT and calcium salts helped to prevent weight loss in strawberry, persimmon, guava, and atemoya fruits, respectively, to a greater extent than the individual treatment application.

**TABLE 1:** Effect of hydrothermal treatment (HT), calcium chloride (CaCl_2_), and their combination (HT‐CaCl_2_) on the weight loss, external color (*L* ^∗^, Hue°), and firmness of papaya cv. Maradol stored at 12°C for 20 days.

Treatments	Days of storage at 12°C					
	0	4	8	12	16	20
*Weight loss (%)*						
Control	—	2.20 ± 0.1^kl^	4.94 ± 0.4^hijk^	7.02 ± 0.9^fghi^	10.47 ± 1.1^bcd^	13.67 ± 1.3^a^
HT	—	2.08 ± 0.2^kl^	4.35 ± 0.6^ijkl^	6.40 ± 0.9^fghij^	8.79 ± 1.3^cdef^	11.1 ± 2.0^abc^
CaCl_2_	—	2.24 ± 0.2^kl^	4.92 ± 0.4^hijk^	7.46 ± 0.7^efgh^	10.32 ± 1.9^bcde^	12.61 ± 2.2^ab^
HT‐CaCl_2_	—	1.70 ± 0.2^l^	3.71 ± 0.6^jkl^	5.57 ± 0.7^ghij^	7.91 ± 1.1^defg^	10.48 ± 1.5^bcd^

*External color (* *L* ^∗^ *)*						
Control	47.69 ± 1.3^hij^	53.13 ± 3.2^fg^	54.52 ± 2.6^df^	58.06 ± 3.9^bcd^	59.52 ± 2.0^bc^	67.07 ± 3.4^a^
HT	46.47 ± 0.9^j^	50.88 ± 3.1^gh^	52.28 ± 4.8^fg^	54.86 ± 5.3^def^	55.08 ± 4.3^def^	60.25 ± 2.2^bc^
CaCl_2_	47.44 ± 5.9^hij^	52.12 ± 2.2^fg^	53.40 ± 2.3^efg^	56.99 ± 3.4^cde^	57.80 ± 6.3^bcd^	61.41 ± 0.8^b^
HT‐CaCl_2_	46.72 ± 4.2^ij^	50.40 ± 5.7^ghi^	53.14 ± 4.4^fg^	54.82 ± 3.7^def^	54.43 ± 0.8^def^	57.55 ± 1.5^cd^

*External color Hue (H°)*						
Control	122.60 ± 4.0^ab^	110.82 ± 4.8^fg^	103.88 ± 3.3^h^	96.77 ± 2.50^jk^	90.79 ± 3.1^lm^	89.96 ± 4.98^m^
HT	122.10 ± 1.4^ab^	117.86 ± 1.9^de^	118.43 ± 2.0^cde^	119.38 ± 1.3^bcd^	116.45 ± 2.5^e^	96.99 ± 2.07^j^
CaCl_2_	124.43 ± 1.7^a^	112.92 ± 5.4^f^	108.51 ± 4.4^g^	101.01 ± 2.9^hi^	93.58 ± 6.5^kl^	93.46 ± 2.23^l^
HT‐CaCl_2_	121.67 ± 1.2^ab^	121.25 ± 0.7^abc^	122.03 ± 1.5^ab^	119.69 ± 2.3^bcd^	120.28 ± 1.0^bcd^	98.99 ± 2.29^ij^

*Firmness (N)*						
Control	65.98 ± 9.9^a^	54.32 ± 8.0^bc^	39.61 ± 15.4^fg^	23.81 ± 3.3^j^	26.14 ± 2.4^ij^	24.88 ± 6.0^j^
HT	66.25 ± 6.6^a^	46.94 ± 10.3^d^	38.56 ± 13.5^fg^	36.34 ± 7.7^fgh^	35.68 ± 5.4^fgh^	29.08 ± 4.2^hij^
CaCl_2_	66.24 ± 7.6^a^	49.98 ± 10.6^cd^	40.98 ± 12.5^f^	32.56 ± 4.8^gi^	35.21 ± 6.8^fgh^	32.76 ± 4.5^gi^
HT‐CaCl_2_	67.57 ± 4.3^a^	57.99 ± 9.8^b^	55.72 ± 8.9^bc^	39.97 ± 5.8^fg^	38.93 ± 7.4^fg^	33.28 ± 5.1^fghi^

*Note:* Values represent the means for three replicates ± standard deviation. For the same parameter, different letters indicate significant differences (*p* < 0.05) among treatments and days.

#### 3.1.2. External Color

The initial luminosity (*L* ^∗^) in the papaya peel was about 46.47–47.69, and these values increased during storage in the four treatments, reaching values between 57 and 67 (Table 1). The increase in *L* ^∗^ coincides with Almeida‐Castro et al. [25], Zerpa‐Catanho et al. [1], and Santamaría‐Basulto et al. [15] in the peel of papaya fruits, and it is associated with a normal ripening process because there is a color change from dark green to orange, increasing *L* ^∗^ due to the presence of lighter colors. At the end of the storage, control fruits had the highest *L* ^∗^, followed by CaCl_2_, while HT and HT‐CaCl_2_ had the lowest values, which may be due to a delay in ripening, decreasing the degradation of chlorophyll and production of carotenoids. Similar results were reported in kiwifruit treated with HT‐CaCl_2_ dips, which maintained *L* ^∗^ values during the storage compared with the rest of the treatments individually [10]. Also, Aguayo et al. [6] found a lower *L* ^∗^ value in apple slices treated with HT than in untreated slices. Contrary to our results, Ayón‐Reyna et al. [16] reported a higher luminosity in papaya fruit treated with HT‐CaCl_2_ indicating that the HT was able to melt the natural wax of the fruit, producing a more homogeneous surface that better reflects light. Although the same cultivar and treatment conditions were used in both studies, these differences in luminosity may have occurred because different stages of ripening were used, and in the previously reported experiment, the fruits were inoculated with the fungus *Colletotrichum gloeosporioides*.

At the beginning of storage, the Hue° values were around 121.67–124.43, which had green tones, and as the days went by these values decreased, changing from green to yellow–orange color. For the last day of storage, control fruits showed the greatest color change because they had the lowest Hue° values followed by CaCl_2_‐treated fruit, while HT and HT‐CaCl_2_ retained the color change longer because they had the highest values. The decrease in Hue° values during the storage is due to the color change from green to yellow–orange in the epidermis of the fruit in response to its natural ripening process, in which chlorophyll gradually degrades and the formation of carotenoid pigments occurs [25]. Likewise, coloration change was delayed by the application of treatments, and this could be due to the effect of high temperatures of HT, favoring an inhibition in the expression of specific genes of maturation, including lycopene synthase [26]. Similarly, Ayón‐Reyna et al. [16] observed that Hue° values of papaya decreased with storage time, and the skin color changed from slightly orange with green stripes to completely orange at the end of the period; likewise, fruits treated with HT‐CaCl_2_ showed the highest values of Hue°, resulting in a lower speed of color change than the control fruits. However, it is important to mention that although the fruits used in that study had a higher maturity index and were inoculated with the fungus *C. gloeosporioides*, the behavior in the color change, in general, was similar to what was found in our study. Moreover, Sharma et al. [27] observed better color retention in apple peel treated with preharvest CaCl_2_ plus postharvest HT than individual treatments. Soliva‐Fortuny et al. [28] found that the application of calcium delayed changes in the color of the surface of the apple fruit. Contrary results were reported by Caleb et al. [29], who observed that the Hue° parameter in strawberry was not affected by the application of HT.

#### 3.1.3. Firmness

The firmness of papaya fruits decreased during storage at 12°C, with significant differences between treatments appearing from the fourth day of storage (*p* < 0.05) (Table 1). Control fruits had the highest firmness loss (about 63%). Fruits treated with HT or CaCl_2_ had similar values throughout storage but presented lower firmness values than those treated with HT‐CaCl_2_, which obtained the highest values during storage. The decrease in firmness is due to the action of ethylene‐induced hydrolytic enzymes, which degrade polymeric carbohydrates, mainly pectins and hemicelluloses, triggering the weakening of the cell wall [1]. Likewise, it is suggested that the delay in the decrease in firmness is influenced by the applied treatments, and it is possible that the pectin methylesterase (PME) enzyme was activated in response to the high temperature of HT. PME can demethylate pectins from the cell wall and the central lamina, favoring the union of endogenous and exogenous calcium with free carboxyl groups, resulting in less polygalacturonase activity and greater firmness [5, 12]. Also, Silveira et al. [13] and Aguayo et al. [6] reported that HT‐CaCl_2_ maintains the texture, and it is generally explained in terms of PME activation. In addition, various authors have pointed out the positive effect of HT when combined with CaCl_2_ on the firmness of whole and fresh‐cut fruits [12–14, 23].

### 3.2. Bioactive Compounds

#### 3.2.1. AA

**FIGURE 1:**
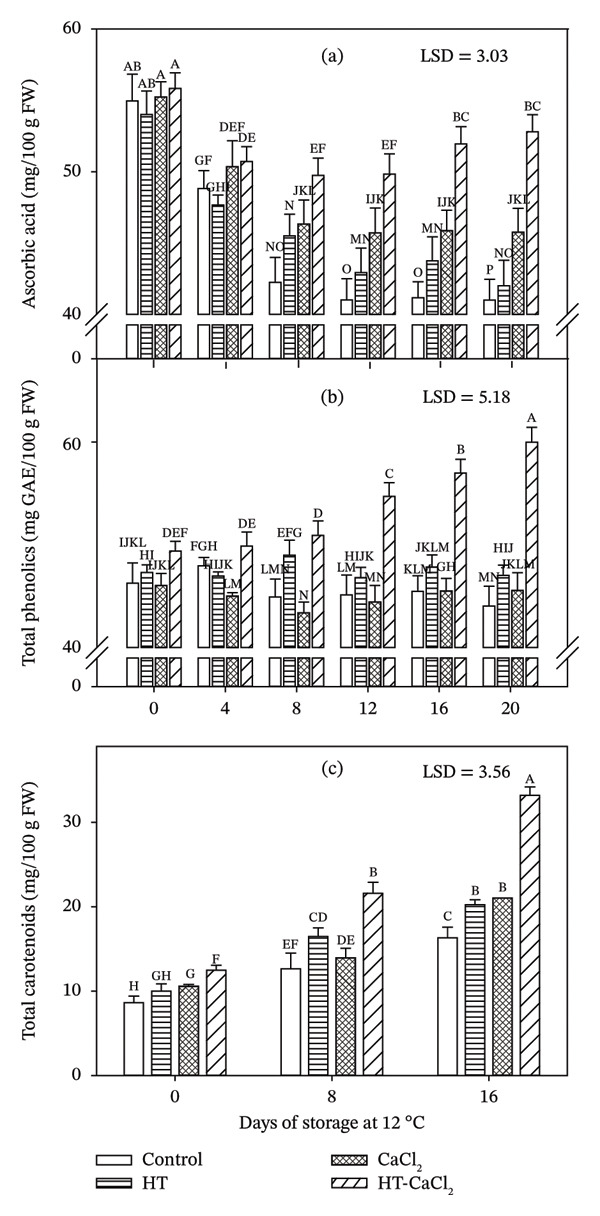
Effect of hydrothermal treatment (HT), calcium chloride (CaCl_2_), and their combination (HT‐CaCl_2_) on the content of ascorbic acid (a), total phenolics (b), and total carotenoids (c) of papaya fruits cv. Maradol stored at 12°C. Vertical bars in the columns represent the standard deviation of the means for three replicates. Different letters indicate significant differences among treatments and days (*p* < 0.05).

The initial concentration of AA was approximately 55 mg AA/100 g FW for all treatments, and during storage the control fruits had the lowest AA content, followed by HT, CaCl_2_, and HT‐CaCl_2_ (Figure 1(a)). Fruits treated with HT‐CaCl_2_ showed a decrease during the first days of storage, followed by an increase in the last days, obtaining the highest concentrations, while HT‐ and CaCl_2_‐treated fruits always tended to decrease or remain the same. The results of this investigation agree with Ayón‐Reyna et al. [12] and Wall [30], who observed an AA content between 50 and 52 mg AA/100 g FW in papaya. Likewise, Acosta‐Ramos et al. [31] pointed out that prolonging the shelf life of papaya fruit resulted in decreased AA content, which could be due to the degradation of AA by a natural process of senescence. The conservation and even the synthesis of AA can be influenced by the application of different treatments, such as the case of Shahkoomahally and Ramezanian [10], who suggested that the application of an HT maintained the content of AA in kiwifruit in comparison with the untreated fruits, which displayed lower contents. Castellano et al. [23] reported that AA content was higher in guava fruits treated with HT at 45°C than in untreated ones. In addition, Aghdam et al. [8] mention that cherry fruits treated with CaCl_2_ presented a higher content of AA compared to control fruits. The effectiveness of the combined treatments (HT calcium salt) has been reported in atemoya (HT 40°C; CaCl_2_ 6%), strawberry (HT 45°C; CaCl_2_ 1%), apple slices (HT 48°C and 55°C; 6% CaCl_2_), and persimmon (HT 45°C and 50°C; 1% calcium lactate [CaLac]), observing higher content compared to control or individual treatments [6, 11, 17, 24]. The decrease in AA is also attributed to the fact that the vitamin is photosensitive and very unstable, being able to easily oxidize to the dehydroascorbic form in the presence of light or heat, as factors such as pH, water activity, and oxygen concentration accelerate the speed of this reaction [32]. In this sense, HT melts the epicuticular wax covering stomata and lenticels, decreasing the entry of oxygen into the fruit cells and therefore reducing the respiration rate and AA oxidation reactions [16]. Also, the integration of calcium into cells as a consequence of the action of HT improves the integrity of the cell wall since it interacts with the cellular matrix of plants due to the formation of bonds between pectins and other components of the cell wall; this reduces tissue permeability and, consequently, AA leaching [33]. In addition to this, it has been observed that the use of HT and CaCl_2_ favors the stability of AA, presumably as a consequence of the inactivation of the enzyme ascorbate oxidase [8, 10].

#### 3.2.2. TP

Papaya fruits treated with HT‐CaCl_2_ showed a higher initial TP content (50 mg GAE/100 g FW) than the other treatments (42–43 mg GAE/100 g FW) (Figure 1(b)). The TP content is in concordance with that reported by Mahattanatawee et al. [34] and Siriamornpun and Kaewseejan [35], who reported a TP content in papaya fruits of 44 and 42 mg GAE/100 g FW, respectively. For their part, Patthamakanokporn et al. [36] reported a TP content of 54 mg GAE/100 g FW in papaya cv. Maradol. On the other hand, a constant behavior could be observed during the 20 days of storage for the control fruits and the individual HT and CaCl_2_ treatments; however, for the fruits treated with HT‐CaCl_2_ from Day 12, a significant increase was observed (*p* < 0.05) and at the end of storage, these fruits presented values above 62 mg GAE/100 g FW, which corresponds to a 24% increase compared to the initial day. These results are similar to those reported by Shahkoomahally and Ramezanian [10] in kiwifruit, Sharma et al. [27] in apples, and Aguayo et al. [6] in fresh‐cut apple. These authors reported a higher TP content in fruits treated with HT combined with calcium salts compared to control fruits and those treated individually with HT or calcium salt. The increase in the TP content that occurred in the fruits treated with the HT‐CaCl_2_ could be the response of the antioxidant system due to the stress to which the fruit was subjected through the heat of the HT treatment, as well as to the positive effect of the calcium, maintaining cellular integrity and preventing the possible contact of the enzyme with its substrate, helping to decrease the degradation activity of the polyphenoloxidase enzyme, keeping this way the content of phenolic compounds stable [10]. TP synthesis during the storage period may be a result of the combination of precursors derived from the shikimate and acetate pathways [37].

#### 3.2.3. TC

The initial concentration of TC in papaya fruits was different among treatments, with values from 8.63 mg of β‐carotene/100 g FW for control fruits to 12.45 mg of β‐carotene/100 g FW for HT‐CaCl_2_‐treated fruits, with intermediate values for individual treatments (HT and CaCl_2_) (Figure 1(c)). These values tended to increase, and by Day 8 the values ranged between 12.65 and 21.60 mg of β‐carotene/100 g FW; at the end of storage, the values ranged from 16.32 to 33.20 mg of β‐carotene/100 g FW. It should be noted that the fruits with the highest TC content during all storage were those treated with HT‐CaCl_2_. The rest of the treatments (HT and CaCl_2_) presented a less pronounced increase compared to the combination, but they had significantly higher values (*p* < 0.05) with respect to the control. The effect of HT has been reported by Glowacz et al. [38], who mention that HT has a positive effect on the TC content in spinach, showing an increase during storage. Likewise, the combination HT‐CaCl_2_ (49°C; 1%) was applied to papaya slices, and an increase in β‐carotene and lycopene was observed during the storage [12]. In addition, Ordóñez et al. [39] reported that the application of HT in fruits and vegetables improves the bioavailability of carotenoids, probably by dissociating protein complexes. Likewise, while the fruits are exposed to heat, the expression of genes of the enzymes that participate in the biosynthesis of carotenoids is promoted; this may indicate the increase in the content of TC during the storage of papaya fruits [39]. The increase in TC content is also related to the fact that during maturation, chlorophyll began to degrade, coinciding with the synthesis of carotenoids, resulting in a significant increase in yellow‐orange color [40].

### 3.3. Antioxidant Capacity

#### 3.3.1. ABTS

**FIGURE 2:**
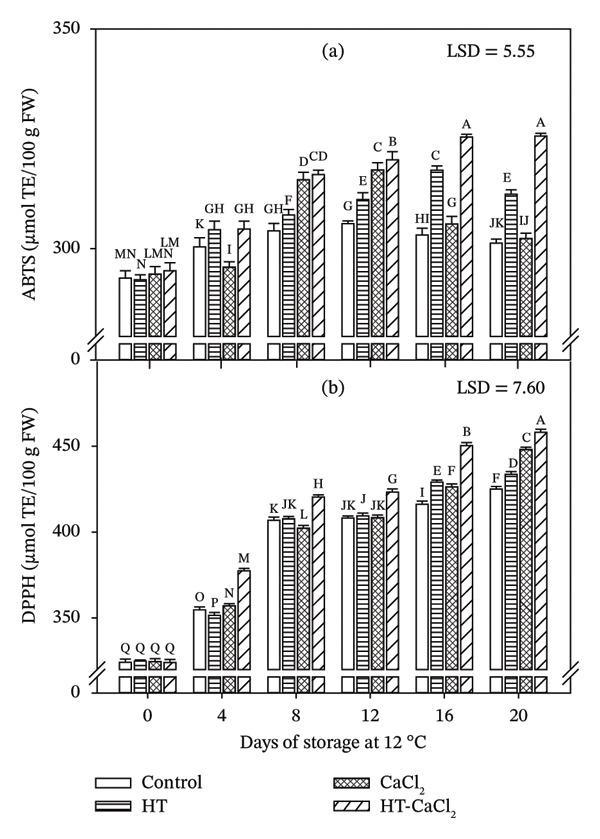
Effect of hydrothermal treatment (HT), calcium chloride (CaCl_2_), and their combination (HT‐CaCl_2_) on the antioxidant capacity measured by ABTS (a) and DPPH (b) methods in papaya cv. Maradol stored at 12°C for 20 days. Vertical bars on the columns represent the standard deviation of the means for three replicates. Different letters indicate significant differences among treatments and days (*p* < 0.05).

The antioxidant capacity values, obtained by the ABTS method, found on the initial day varied between 292.93 and 294.94 μmol TE/100 g FW, showing the lowest values in fruits treated with HT and the highest values in fruits treated with HT‐CaCl_2_. Subsequently, antioxidant capacity increased during the storage, and at the end of it, the antioxidant capacity ranged from 301.23 μmol TE/100 g FW for control fruits to 325.62 μmol TE/100 g FW for HT‐CaCl_2_‐treated fruits (Figure 2(a)). Despite CaCl_2_ treatment showing higher antioxidant capacity than control during Days 8, 12, and 16, at the end of the storage, no significant differences were observed between these treatments. The application of HT, CaCl_2_, and HT‐CaCl_2_ treatments influenced the antioxidant capacity of the hydrophilic compounds present in papaya fruits, which interact with the ABTS radical [41]. The papaya fruits evaluated in this study had a higher antioxidant activity than those evaluated by Leong and Shui [42] in the papaya fruit variety Solo (141 μmol TE/100 g FW) as well as in the papaya fruit variety Foot Long (72.50 μmol TE/100 g FW), both in a state of commercial maturity.

#### 3.3.2. DPPH

The antioxidant capacity measured by the DPPH method is shown in Figure 2(b). The initial concentration was between 324.04 and 324.84 μmol TE/100 g FW, without showing significant differences among treatments; later, the activity increased to finish the storage with values between 425.10 and 458.13 μmol TE/100 g FW. Significant differences (*p* < 0.05) were observed between all treatments, having control fruit the lowest values, followed by HT, CaCl_2_, and HT‐CaCl_2_, being the last treatment the one with the highest values. The increase in antioxidant capacity observed during storage may be associated with the presence and maintenance of antioxidant compounds such as phenolics and AA in papaya fruit. These compounds contribute significantly to the radical scavenging activity measured by ABTS and DPPH assays, as they are capable of neutralizing ROS. Therefore, the higher antioxidant capacity observed in HT‐CaCl_2_‐treated fruits could be related to the better preservation and accumulation of these bioactive compounds during storage. In previous reports, Beserra‐Almeida et al. [43] obtained an antioxidant capacity by the DPPH method of 224 μmol TE/100 g FW in papaya fruit; likewise, Gayosso‐García et al. [44] reported an antioxidant activity by the DPPH method of 116 μmol TE/100 g FW in papaya pulp cv. Maradol in state of maturity 4. Also, similar results were observed by Caleb et al. [29], where the antioxidant capacity in strawberries determined by the DPPH increased during storage. The increased antioxidant capacity in the fruits may be a response to the synergistic effect of the combination of the treatments, since the temperature of HT can raise the speed of calcium diffusion in the fruit tissue, improving its structure. In addition to this, treatments can increase the solubility of bioactive compounds in water [6]. Aguayo et al. [6] reported that the application of an HT together with calcium ascorbate generated a greater capacity to eliminate oxidative agents, presenting significant differences (*p* < 0.05) between apple fruits treated with the combination regarding control. Aghdam et al. [8] reported that the application of CaCl_2_ in cherry fruits allowed to obtain a higher antioxidant capacity by the DPPH method in the treated fruits compared to the control fruits. Increased antioxidant activity helps reduce oxidation, which slows down the degradation of compounds such as sugars, organic acids, and vitamins (e.g., vitamin C). Also, when the antioxidant system is activated and the antioxidant capacity of the fruit increases, an improvement in the conservation of the physicochemical quality of the fruit is generally observed, which is consistent with the results obtained in this study, since the untreated fruits presented the lowest antioxidant capacity and the greatest loss of quality, while fruits treated with HT‐CaCl_2_ presented the highest antioxidant capacity and the greatest retention of physicochemical quality.

### 3.4. Enzymatic Activity

#### 3.4.1. POD

**FIGURE 3:**
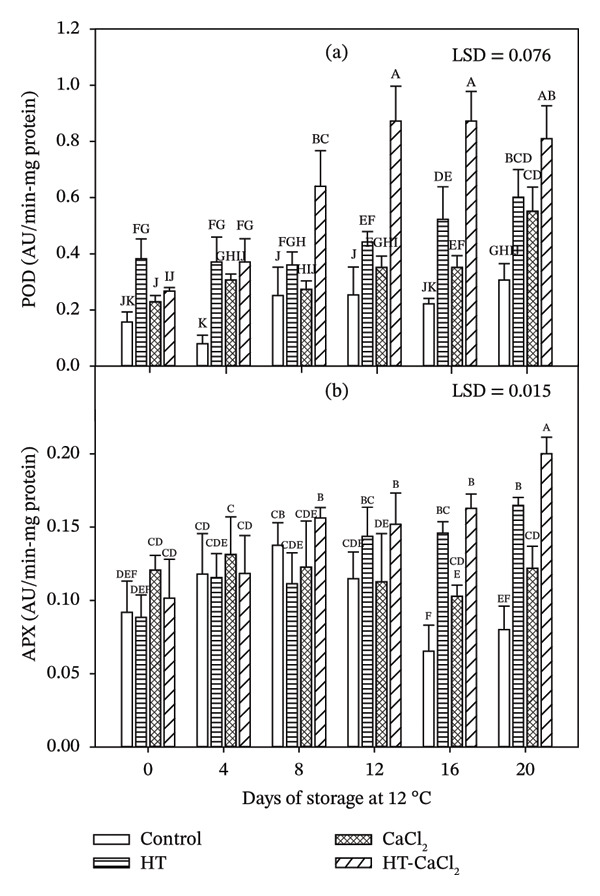
Effect of hydrothermal treatment (HT), calcium chloride (CaCl_2_), and their combination (HT‐CaCl_2_) on the antioxidant activity of peroxidase (POD) (a) and ascorbate peroxidase (APX) (b) in fruits of papaya cv. Maradol stored at 12°C for 20 days. Vertical bars on the columns represent the standard deviation of the means for three replicates. Different letters indicate significant differences among treatments (*p* < 0.05).

POD enzyme controls the levels of H_2_O_2_ that are generated in almost all living cells, so its action is very important for plants since it avoids the damaging effect of free radicals [45]. According to the results obtained in this study, POD activity was influenced by the applied treatments since in the control fruits no significant changes were detected during the storage time, while in the treated fruits an increase in the activity was observed (*p* < 0.05) (Figure 3(a)). In fruits treated with HT and CaCl_2_ the enzyme activity remained unchanged during the first 12 days of storage, with values close to 0.40 and 0.30 UA/min‐mg protein, respectively; however, from that day on, activity increased, reaching its maximum point at the end of storage. For HT fruits, a value of 0.60 UA/min‐mg protein was obtained, and a value of 0.55 UA/min‐mg protein for those treated with CaCl_2_. In the fruits treated with HT‐CaCl_2_, the activity of POD was increasing from the 4th day of storage with a value of 0.37 UA/min‐mg protein; later, its maximum activity was reflected on Day 12 of storage with a value of 0.87 UA/min‐mg protein, which remained unchanged for the rest of the storage. The increase in activity in response to the exposure of the fruit to the applied treatments may be due to the activation of latent forms or new isoforms of PODs, solubilization of those that were bound to the cell wall, or de novo synthesis present in the cells of papaya fruits [46]. Likewise, it is reported that the observed increase may be associated with the oxidative stress caused by the exposure of the fruits to unfavorable temperature conditions, so that a greater release of POD to the cytoplasm can be generated, increasing, in this way, its activity [47]. Also, it may be due to lignification in the cell walls of fruits because Ca^+2^ induces the crosslinking of polygalacturonan chains, forming a structure that can be recognized by isoperoxidase [48]. Similar results were obtained in atemoya fruit with the application of HT at 40°C in combination with 6% CaCl_2_, which favored the POD activity during the first days of storage [24]. For their part, Zhou et al. [49] mentioned that the application of HT increased POD activity in orange fruits stored at 20°C for 35 days, while Boonkorn [46] indicated that exposure of tomato fruits to HT (35°C and 40°C) had an influence on POD activity, noting that as the temperature increased, the enzyme activity also did. Likewise, Ghasemnezhad et al. [50] pointed out that the higher the applied temperature (HT at 45°C, 47.5°C, 50°C, 52.5°C, and 55°C) to mandarin fruits, the enzymatic activity of POD was higher, obtaining significant differences between the treatments in comparison with the control fruits.

#### 3.4.2. APX

The APX enzyme is involved in the ascorbate–glutathione cycle, which uses ascorbate as an electron donor, and plays the most essential role in the capture of ROS; therefore, it is used as an essential element in the response to oxidative stress caused in plants [51]. HT and HT‐CaCl_2_‐treated fruit showed an increase in APX activity during the complete storage, while CaCl_2_‐treated fruit remained constant and control fruit showed an increase during the first days and a decrease the last days of storage. At the beginning of storage, all treatments presented similar activities, but, from Day 12, significant differences (*p* < 0.05) were found between HT‐CaCl_2_ and the CaCl_2_ and control fruits; likewise, on Days 16 and 20, differences were reflected (*p* < 0.05) between CaCl_2_ and control, as well as HT and control (Figure 3(b)). According to Imahori et al. [51], the increases in APX activity would contribute to the elimination of hydrogen peroxide, effectively eliminating ROS, thereby protecting cellular components from the highly reactive hydroxyl radical. The decreasing trend of APX activity observed in the last days of storage in untreated papaya was similar to that of mume fruit stored for 16 days at 6°C [51]. For their part, Shadmani et al. [7] observed that the application of a double HT, first at 42°C for 30 min and then at 49°C for 20 min, in papaya cv. Franji increased the enzymatic activity of APX during the storage at 12°C. The increase in the enzymatic activity of APX may be the result of a thermal tolerance caused by the heat of the HT. As a result, the moderate stress generated activated cellular signaling pathways and cellular response, followed by the production of oxygen radical scavengers such as APX [52]. Likewise, heat stress can affect the ability of biological systems to synthesize proteins, resulting in the synthesis of a new set of special proteins called heat shock proteins (HSP), which manifest in most living organisms as induction or enhanced synthesis of HSPs. These proteins are known to prevent denaturation and irreversible degradation of the protein, which would be detrimental to the cell, in addition to conferring heat tolerance [53].

The increase in POD and APX activities observed under the combined HT‐CaCl_2_ treatment may be associated with the activation of the fruit antioxidant defense system in response to postharvest stress. HTs can act as mild abiotic stressors that stimulate protective metabolic responses, including the activation of antioxidant enzymes involved in the detoxification of ROS. In this context, POD and APX play key roles in the scavenging of hydrogen peroxide and the maintenance of cellular redox balance, thereby contributing to the reduction of oxidative damage during storage [9]. Additionally, calcium ions are known to stabilize cell membranes and cell wall structures, which may help preserve cellular compartmentalization and enzyme functionality. Calcium has also been reported to participate in signaling pathways associated with stress responses, potentially enhancing the activity of antioxidant enzymes. Therefore, the combined application of HT and CaCl_2_ may exert a synergistic effect by simultaneously inducing antioxidant defense mechanisms and improving structural stability in fruit tissues [5]. Similar responses have been reported in other tropical fruits subjected to postharvest treatments aimed at reducing oxidative stress. For instance, increased activities of antioxidant enzymes such as POD or APX have been observed in mango [5, 9], papaya [7], and apple [14] following heat treatments or calcium applications, which were associated with improved oxidative stability and delayed senescence.

Activation of antioxidant enzymes can help preserve bioactive compounds during fruit storage, thereby maintaining their nutritional value. As observed in this study, the fruits treated with HT‐CaCl_2_ presented the highest POD and APX activity and the highest content of AA, phenols, and carotenoids, in addition to presenting the greatest retention of physicochemical quality. These findings support the hypothesis that the enhancement of antioxidant enzyme systems represents an important mechanism underlying the preservation of fruit quality during postharvest storage.

## 4. Conclusions

HT‐CaCl_2_ treatment can act as a stimulator of the natural antioxidant system of papaya fruit cv. Maradol by improving the content of bioactive compounds, the antioxidant capacity, and the antioxidant enzymatic activity, representing an alternative process for its conservation because this treatment showed to be effective to preserve the postharvest quality. From a practical perspective, the integration of HT with CaCl_2_ represents a promising postharvest technology that could be applied at the commercial level to extend shelf life and maintain fruit quality during storage and distribution. Such treatments may provide an accessible and relatively low‐cost alternative for improving postharvest management in fresh fruit supply chains. For this reason, HT‐CaCl_2_ may be a useful strategy to extend the shelf life and promote the activation of the antioxidant system in horticultural products.

## Funding

No funding was received for this manuscript.

## Conflicts of Interest

The authors declare no conflicts of interest.

## Data Availability

The data that support the findings of this study are available from the corresponding author upon reasonable request.
